# Supplementary material for: Post-correlation on-lamella cryo-CLEM reveals the membrane architecture of lamellar bodies
Source: Commun Biol. 2021 Jan 29;4:137. doi: 10.1038/s42003-020-01567-z (PMC7846596; doi:10.1038/s42003-020-01567-z)
Supplement: Supplementary file 2 — Descriptions of Additional Supplementary Files [file 42003_2020_1567_MOESM2_ESM.pdf]

Descriptions of Additional Supplementary Files

Supplementary movie 1

Description: Tomogram of LB organelles showing parallel-curved membrane packaging of membrane sheets and crystalline lipid structures (corresponding to Figure 3).

Supplementary movie 2

Description: Tomogram and rendering of LB-like organelle showing membrane sheet termini and “T”-junctions (corresponding to Figure 4).

Supplementary movie 3

Description: Tomogram of LB-like organelle showing membrane sheet termini and “T”-junctions (corresponding to Figure 4).

Supplementary Data 1

Description: Summary of measured values and statistics used in the study.
