# Supplementary material for: Post-correlation on-lamella cryo-CLEM reveals the membrane architecture of lamellar bodies
Source: Commun Biol. 2021 Jan 29;4:137. doi: 10.1038/s42003-020-01567-z (PMC7846596; doi:10.1038/s42003-020-01567-z)
Supplement: Supplementary file 7 — Reporting Summary [file 42003_2020_1567_MOESM7_ESM.pdf]

nature research

Corresponding author(s): Petr Chlanda

Last updated by author(s): Nov 6, 2020

Reporting Summary

Nature Research wishes to improve the reproducibility of the work that we publish. This form provides structure for consistency and transparency in reporting. For further information on Nature Research policies, see our Editorial Policies and the Editorial Policy Checklist.

Statistics

For all statistical analyses, confirm that the following items are present in the figure legend, table legend, main text, or Methods section.

n/a	Confirmed	
☐	☒	The exact sample size (*n*) for each experimental group/condition, given as a discrete number and unit of measurement
☐	☒	A statement on whether measurements were taken from distinct samples or whether the same sample was measured repeatedly
☒	☐	The statistical test(s) used AND whether they are one- or two-sided<br>*Only common tests should be described solely by name; describe more complex techniques in the Methods section.*
☒	☐	A description of all covariates tested
☒	☐	A description of any assumptions or corrections, such as tests of normality and adjustment for multiple comparisons
☐	☒	A full description of the statistical parameters including central tendency (e.g. means) or other basic estimates (e.g. regression coefficient) AND variation (e.g. standard deviation) or associated estimates of uncertainty (e.g. confidence intervals)
☒	☐	For null hypothesis testing, the test statistic (e.g. *F*, *t*, *r*) with confidence intervals, effect sizes, degrees of freedom and *P* value noted<br>*Give P values as exact values whenever suitable.*
☒	☐	For Bayesian analysis, information on the choice of priors and Markov chain Monte Carlo settings
☒	☐	For hierarchical and complex designs, identification of the appropriate level for tests and full reporting of outcomes
☒	☐	Estimates of effect sizes (e.g. Cohen's *d*, Pearson's *r*), indicating how they were calculated

Our web collection on statistics for biologists contains articles on many of the points above.

Software and code

Policy information about availability of computer code

Data collection	SerialEM, Leica cryo-LM software, MAPS
Data analysis	IMOD, FIJI, MATLAB, ICY, AutoQuant X3, Dynamo, Imaris 9.5.1. The FIJI/ImageJ macro for fluorescent map assembly and the MATLAB code used for 3D stack registration are. available on github under https://github.com/chlanda-lab.

For manuscripts utilizing custom algorithms or software that are central to the research but not yet described in published literature, software must be made available to editors and reviewers. We strongly encourage code deposition in a community repository (e.g. GitHub). See the Nature Research guidelines for submitting code & software for further information.

Data

Policy information about availability of data

All manuscripts must include a data availability statement. This statement should provide the following information, where applicable:

- Accession codes, unique identifiers, or web links for publicly available datasets
- A list of figures that have associated raw data
- A description of any restrictions on data availability

EMD-10818, EMD-10820, EMD-10821, EMD-10819, EMD-11929, EMD-11931

Field-specific reporting

Please select the one below that is the best fit for your research. If you are not sure, read the appropriate sections before making your selection.

[x] Life sciences [ ] Behavioural & social sciences [ ] Ecological, evolutionary & environmental sciences

For a reference copy of the document with all sections, see nature.com/documents/nr-reporting-summary-flat.pdf

Life sciences study design

All studies must disclose on these points even when the disclosure is negative.

Sample size	Sample size estimation was not performed. The workflow was optimized on several trials. We classified structures corresponding to ABCA3-eGFP isolated signals (n = 62) on 6 lamellae.
Data exclusions	ryo-lamellae that showed devitrifications were not used for the data analysis.
Replication	The presented data are coming from two independent biological replicates.
Randomization	The cells were selected for cryo-FIB milling based on the fluorescent signal and a suitable cell position on the grid (center of the square) and therefore a randomized selection is not possible. However, cell seeding results in a stochastically distributed cells on the EM grids.
Blinding	Blinding was not used in this study but most of the quantifications and measurements were confirmed independently by another person. Since the aim of the study was to describe the morphology of lamellar bodies and this project was not hypothesis driven but mainly descriptive we did not implemented blinding into experimental design.

Reporting for specific materials, systems and methods

We require information from authors about some types of materials, experimental systems and methods used in many studies. Here, indicate whether each material, system or method listed is relevant to your study. If you are not sure if a list item applies to your research, read the appropriate section before selecting a response.

Materials & experimental systems

n/a	Involved in the study	
[x]	[ ]	Antibodies
[ ]	[x]	Eukaryotic cell lines
[x]	[ ]	Palaeontology and archaeology
[x]	[ ]	Animals and other organisms
[x]	[ ]	Human research participants
[x]	[ ]	Clinical data
[x]	[ ]	Dual use research of concern

Methods

n/a	Involved in the study	
[x]	[ ]	ChIP-seq
[x]	[ ]	Flow cytometry
[x]	[ ]	MRI-based neuroimaging

Eukaryotic cell lines

Policy information about cell lines

Cell line source(s)	HSAEpC primary human small airway epithelial cells were purchased from PromoCell (Lot 414Z012.2); A549 cells were purchased from ECACC (Lot 16J012)
Authentication	None of the cell lines were authenticated.
Mycoplasma contamination	All cells were tested negative for mycoplasma contamination using the mycoalert kit (Lonza, cat# LT07-118).
Commonly misidentified lines (See ICLAC register)	No commonly misidentified lines were used.
